# Gene expression analysis of membrane progesterone receptors in women with recurrent spontaneous abortion: a case control study

**DOI:** 10.1186/s13104-019-4787-x

**Published:** 2019-12-04

**Authors:** Reyhane Rahnama, Mitra Rafiee, Saloomeh Fouladi, Maryam Akbari-Fakhrabadi, Ferdos Mehrabian, Abbas Rezaei

**Affiliations:** 1 Department of Immunology, School of Medicine, Isfahan University of Medical Sciences, Isfahan, Iran; 2 Department of Nutrition, School of Public Health, Iran University of Medical Sciences, Tehran, Iran; 3 Department of Obstetrics and Gynecology, Al-Zahra Hospital, Isfahan University of Medical Sciences, Isfahan, Iran

**Keywords:** RSA, Progesterone, *mPR*, Endometrium, *NPR*

## Abstract

**Objective:**

Recurrent spontaneous abortion (RSA) is a condition which is defined as three consecutive pregnancy losses prior to 20 weeks from the last menstrual period. Progesterone is a steroid hormone that has an essential role in the implantation and maintenance of pregnancy. The progesterone signaling is performed by nuclear progesterone receptors (NPRs) and membrane progesterone receptors (mPR). The aim of this study was to analyze gene expression of *mPR*-*α*, *mPR*-*β* and NPR in the endometrium of patients with a history of RSA compared to normal fertile women.

**Results:**

In this study, endometrial samples were obtained from 10 women with a history of RSA and 10 fertile women during days 10–14 of menstrual cycle. Relative expression of *mPR*-*α*, *mPR*-*β* and *NPR* genes were studied by a quantitative real time polymerase chain reaction (qRT-PCR) and compared between the two groups. The mean relative expression of *mPR*-*β* gene was significantly lower in the case group compared to the fertile women (p < 0.05). However, the gene expression of *mPR*-*α* and *NPR* showed no significant difference between two groups. The findings suggest a reduction of endometrial gene expression of *mPR*-*β* in RSA patients may play an important role in pathogenesis of RSA.

## Introduction

Recurrent spontaneous abortion is one of the complications during pregnancy which occurs among 2–5% of couples [1, 2]. This condition is defined as three consecutive pregnancy losses prior to 20 weeks from the last menstrual period. Various factors are involved in the occurrence of abortion which include genetic, anatomical abnormalities of the uterus and also thrombophilic, endocrine, environmental, infectious and immunological factors [3, 4].

However, the cause of RSA remains un-known in around 50% of the patients. Since human endometrium is considered an important determining factor in fertility, it has been proposed that an unsuitable endometrium could be an effective factor leading to RSA [5].

Progesterone is a steroid hormone that is primarily secreted under influences of human chorionic gonadotropin (hCG) by corpus luteum [6]. This hormone has an essential role in reproduction which is involved in the menstrual cycle, implantation and maintenance of pregnancy. Progesterone insufficiently in the luteal phase and early pregnancy could be one of the causes of RSA [7]. Progesterone has an important immunomodulatory role during pregnancy by regulating mother immune responses in preventing fetal rejection. The progesterone physiologic effects are mediated by an interaction with its receptors called progesterone receptors (PRs) [8].

The interaction of progesterone with PRs at the decidua level plays an important role in regulating the maternal immune responses [9]. The Progesterone receptor signaling is performed by both genomic and non-genomic pathways. The genomic pathway is related to nuclear progesterone receptors (NPRs) and the non-genomic pathway is related to membrane receptors (mPR) such as mPR-α and mPR-β that bind progesterone at the cell surface and rapidly generate intracellular second messengers [10]. Furthermore, the other mechanism of progesterone in maintaining pregnancy is declining the uterine contractility and improving the utero-placental blood circulation [11, 12].

Therefore, the therapeutic application of progesterone is targeted to prevent pregnancy complications such as recurrent miscarriage [12]. There is yet controversy in the usage of this clinical method. Some studies have indicated the benefit of progesterone in treatment of RSA [13, 14]; whereas other studies revealed negative results. The latter assert that the inefficiency of progesterone is due to its responsiveness rather than its availability. In fact, in these cases the expression or function of progesterone receptors is involved [15]. A study accomplished in a PR knockout mice model showed that mutation in PR represented defects in all reproductive organs. This included a dysfunction in ovulation, hyperplasia and inflammation in uterine, defect in mammary gland, and incapability in sexual performance [16]. In addition, some specific PR polymorphisms have been reported in women with RSA [17, 18].

Despite the importance of progesterone receptors in determining the correct function of this hormone in preserving pregnancy, so far, no study has been done to investigate the role of these receptors in abortion. Therefore, considering the importance of progesterone in preserving pregnancy and confirming its interaction with its membrane receptors, this study aimed to evaluate for the first time the gene expression of progesterone membrane receptors in endometrial tissue of RSA patients compared to normal controls.

## Main text

### Materials and methods

#### Study population

Our study population included women who were referred to Isfahan Shahid Beheshti Hospital that had regular menstruation cycle and no hormonal, anatomical and chromosomal disorders. All subjects were matched for age and BMI. Women over 42 years old, BMI more than 25, hormonal drug usage during the last 3 months before the sampling, and any gynecological or autoimmune diseases were excluded from our study.

Endometrial samples were taken from both groups during the late proliferative phase of the menstrual cycle (days 10–14) by Pipelle Endometrial Suction Curette (Cooper Surgical Medical Devices, USA). A written consent form approved by the ethical committee of Isfahan University of medical sciences was obtained from all participants.

All procedures performed in studies involving human participants were in accordance with the Ethics Committee of Isfahan University of Medical Sciences (Code of Ethics: IR.MUI.REC.1395.3.057) and with the Helsinki declaration and its later amendments or comparable ethical standards.

#### Recurrent spontaneous abortion cases

The case group included patients with at least two consecutive incidents of miscarriage prior to the 20th week of gestation with no identifiable cause which had been aborted for 3–6 months. All the cases were diagnosed with RSA; being previously evaluated for anatomical, chromosomal, genetic and hormonal abnormalities which had no detectable disorder.

#### Control group

Women who had at least one successful term pregnancy and visited for routine gynecological checkup diagnosed with no specific disorder, or who had undergone operations for unrelated procedures were included in our study as normal controls.

#### RNA isolation and cDNA synthesis

Endometrial samples were washed with Phosphate-Buffered Saline (PBS) and immediately stored in RNAlater (Sigma, USA) in − 20 °C. After defrosting the frozen samples, tissues were removed from RNAlater and then total RNA was extracted using MN NucleoSpin^®^ RNA kit (MACHEREY–NAGEL, Germany) according to the Kit instructions. Thereafter, cDNA synthesis was conducted by using the RevertAid First Strand cDNA Synthesis kit (Thermo Fisher, USA) according to the kit protocol. Consequently the cDNA was then kept at − 20 °C.

#### Quantitative real time PCR (qRT-PCR)

Quantitative real-time PCR (qRT-PCR) was performed by the BioFACT™ 2X real-time PCR Master Mix (Biofact, Korea) on the cDNA samples by an Applied Biosystems StepOne ™ machine (ABI Step One, CA, USA). The primers were designed by the Allele ID 7.0 software (Premier Biosoft, USA) which are listed in Table 1. Amplification was performed under the following conditions: 15 min at 95 °C, 45 cycles of 95 °C for 15 s and 60 °C for 60 s. For all genes a negative control consisting of non-template water instead of cDNA was used in each run of qRT-PCR. The relative quantitative gene expression was normalized by GAPDH, the internal control gene. Furthermore, the 2^−ΔΔCt^ equation was considered for the calculation of relative mRNA levels.

**Table 1:** Sequences of primers used in real time-PCR

Gene	Forward primer	Reverse primer
GAPDH	5′-GAAATCCCATCACCATCTTCCA-3′	5′-CAAATGAGCCCCAGCCTTC-3′
MPRα	5′-CTGAAGTTTGCCTGACACCA-3′	5′-AATAGAAGCGCCAGGTCTGA-3′
MPRβ	5′-CACGAAGGACCCACAAAACT-3′	5′-CAATCCCAAGCACCACCAT-3′
NPR	5′-GCTACGAAGTCAAACCCAGT-3′	5′-CACCATCCCTGCCAATATC-3′

*GAPDH* glyceraldehyde-3-phosphate dehydrogenase, *MPRα* membrane progesterone receptor α, *MPRβ* membrane progesterone receptor β, *NPR* nuclear progesterone receptor

#### Statistical analysis

The data was analysed by SPSS 24 software (IBM, Chicago, IL, USA). The Shapiro–Wilk test was used for evaluating the normal distribution of quantitative data. The genomic factors were analysed by the non-parametric Mann–Whitney test. p value less than 0.05 was considered statistically significant in this study.

### Results

In this study, 10 RSA patients and 10 fertile healthy women were participated. Demographic and clinical characteristics of two groups are presented in Table 2. No significant difference was detected for age and body mass index between the two study groups (BMI, p > 0.05).

**Table 2:** Demographic and clinical characteristic of RSA and normal group

Variable	RSA patients (n = 10)	Healthy controls (n = 10)
Age (years)	31.9 ± 1.32	32.9 ± 1.12
Number of abortion	3.2 ± 2.42	0
Number of successful pregnancies	0	2.82 ± 1.23
BMI (kg/m^2^)	25.6 ± 0.98	26.8 ± 2.14

*BMI* body mass index, *RSA* recurrent spontaneous abortion

**Fig. 1:**
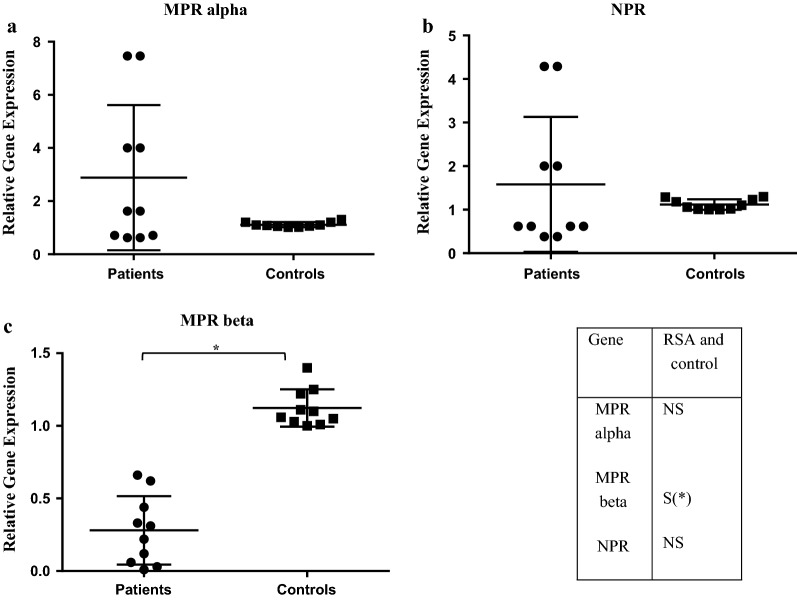
Progesterone receptor genes expression levels. qRT-PCR analysis of **a** MPR alpha, **b** NPR, and **c** MPR beta genes expression levels in the endometrial samples of RSA patients (n = 10) compared to control group (n = 10). Data are presented as mean ± SD, *p < 0.05. *qRT-PCR* quantitative real time PCR, *MPRα* membrane progesterone receptor α, *NPR* nuclear progesterone receptor, *MPRβ* membrane progesterone receptor β, *RSA* recurrent spontaneous abortion, *NS* non-significant, *S* significant

The results of this study showed that *mPR*-*α* gene was expressed higher in endometrium of the RSA group but this finding was not statistically significant compared to the control group (Fig. 1a). In addition *NPR* gene expression did not significantly differ between two study groups (Fig. 1b).

The results of qRT-PCR revealed that the mean relative expression of *mPR*-*β* gene was significantly lower in endometrium of women with RSA compared to normal fertile women (Fig. 1c).

### Discussion

Our study showed that the expression of progesterone membrane receptor (mPR-β) in the endometrial tissue of patients with recurrent spontaneous abortion was significantly lower in comparison with the normal control group. Progesterone hormone is an important steroid hormone which plays an important role in maintaining pregnancy. Progesterone activity depends on progesterone receptors (PRs) which include nuclear progesterone receptors (NPRs) and membrane progesterone receptors (mPRs) [19, 20]. Progesterone hormone plays an important role in the implantation process and maintenance of pregnancy. Therefore, its deficiency and a diminished luteal phase may result in disturbances in endometrium development which is related to RSA. However, a number of studies have demonstrated that progesterone supplementation for RSA patients does not improve pregnancy outcomes in some cases [14, 21].

As mentioned before, it is asserted that the problem is not just the hormone availability; but the abnormality of PRs is also involved. Decreased *PR* expression by the embryo and the endometrium has been associated with RSA [15]. Su et al. reported a specific *PR* polymorphisms in women with a history of RSA [12, 17]. Furthermore, a correlation between RSA and polymorphism in intron G of the PR gene is related to implantation failure [22]. Due to the importance of PRs in progesterone therapy in avoiding preterm birth and recurrent spontaneous abortion [23], the better understanding of PR function in pregnancy complications will be helpful in better diagnosis and therapies in this context [19]. Studies show that the expression of *mPR*-*α* and *mPR*-*β* (two isotypes of *mPRs*) modifies during pregnancy in human endometrium. A decrease in expression of *mPR*-*α* during preterm and term labors has been determined, whereas *mPR*-*β* expression decreases only in term labor [24, 25]. *mPR*-*α* and *mPR*-*β* activate the p38 MAPK signaling pathway and induce phosphorylation which down regulate *SRC2* expression at the end of pregnancy and onset of delivery [25, 26]. In this regard, other mechanisms include Ca^2+^ mobilization, opening of Na^+^ and Cl^−^ channels and the activation of phospholipase C which is involved in *mPR* activation process [27, 28].

Based on our knowledge, this is the first study to compare the expression of progesterone receptors in women suffering from recurrent spontaneous abortion with normal subjects. As previously mentioned, the results of our study showed a decrease in the gene expression of *mPR*-*β* in women with RSA compared to healthy subjects and if this problem occurs during pregnancy, it is likely to affect the normal pregnancy process. In addition, one of the routine treatments implied in RSA patients is progesterone therapy, and insufficiency of progesterone receptors may pose a problem with the treatment process.

### Conclusion

The data of the present study suggest that reduction in expression of *mPR*-*β* is likely to contribute to the etiology of RSA. However, the definite role of membrane progesterone receptors in pathogenesis of RSA needs to be better investigated.

## Limitation

There were some limitations in our study that are suggested to be addressed in future studies in order to better understand the role and function of progesterone membrane receptors. These limitations include: (1) limited number of samples, (2) our study was limited to the late proliferative phase. Since the expression of different progesterone receptors changes during different days of the menstrual cycle, studying the expression of these receptors in other phases of the menstrual cycle is important to better understand their function. (3) Our study has been carried out only at the gene expression level; evaluation of protein expression will show more accurate results. (4) Evaluating the expression of these receptors in the peripheral blood of people with recurrent abortions and comparing them with normal people can also provide valuable information.

## Data Availability

The datasets used and analyzed during the current study are available from the corresponding author on a reasonable request.

## Abbreviations

RSA: recurrent spontaneous abortion
NPRs: nuclear progesterone receptors
mPR: membrane progesterone receptors
qRT-PCR: quantitative real time polymerase chain reaction
hCG: human chorionic gonadotropin
PRs: progesterone receptors
